# A Bayesian elicitation of veterinary beliefs regarding systemic dry cow therapy: Variation and importance for clinical trial design

**DOI:** 10.1016/j.prevetmed.2012.01.017

**Published:** 2012-09-15

**Authors:** H.M. Higgins, I.L. Dryden, M.J. Green

**Affiliations:** a School of Mathematical Sciences, University of Nottingham, University Park Campus, Nottingham NG7 2RD, UK; b School of Veterinary Medicine and Science, University of Nottingham, Sutton Bonington Campus, Sutton Bonington LE12 5RD, UK; c Department of Statistics, LeConte College, University of South Carolina, Columbia, SC 29208, USA

**Keywords:** Probabilistic elicitation, Bayesian analysis, Dry cow therapy, Systemic antibiotics

## Abstract

The two key aims of this research were: (i) to conduct a probabilistic elicitation to quantify the variation in veterinarians’ beliefs regarding the efficacy of systemic antibiotics when used as an adjunct to intra-mammary dry cow therapy and (ii) to investigate (in a Bayesian statistical framework) the strength of future research evidence required (in theory) to change the beliefs of practising veterinary surgeons regarding the efficacy of systemic antibiotics, given their current clinical beliefs.

The beliefs of 24 veterinarians in 5 practices in England were quantified as probability density functions. Classic multidimensional scaling revealed major variations in beliefs both within and between veterinary practices which included: confident optimism, confident pessimism and considerable uncertainty. Of the 9 veterinarians interviewed holding further cattle qualifications, 6 shared a confidently pessimistic belief in the efficacy of systemic therapy and whilst 2 were more optimistic, they were also more uncertain. A Bayesian model based on a synthetic dataset from a randomised clinical trial (showing no benefit with systemic therapy) predicted how each of the 24 veterinarians’ prior beliefs would alter as the size of the clinical trial increased, assuming that practitioners would update their beliefs rationally in accordance with Bayes’ theorem.

The study demonstrated the usefulness of probabilistic elicitation for evaluating the diversity and strength of practitioners’ beliefs. The major variation in beliefs observed raises interest in the veterinary profession's approach to prescribing essential medicines. Results illustrate the importance of eliciting prior beliefs when designing clinical trials in order to increase the chance that trial data are of sufficient strength to alter the clinical beliefs of practitioners and do not merely serve to satisfy researchers.

## 1 Introduction

Quantifying the diversity and strength of practitioners’ clinical beliefs is important for clinical trial design. Conducting a randomised clinical trial (RCT) takes considerable time, effort and expense and hence researchers who are prepared to make this commitment are almost invariably enthusiastic about the trial's outcome at the outset. Their enthusiasm may be markedly different to that of the end consumers of the data (e.g. veterinary practitioners), who may hold very different beliefs, even extreme scepticism. However RCTs that have been designed using traditional frequentist sample size calculations do not (and indeed cannot in this classical statistical framework) take into account the current beliefs of the end consumers of the data. Thus in the event the trial does yield a positive result, this may prove sufficient evidence to convince the researchers themselves, but be of insufficient strength to alter the clinical beliefs of practitioners. Inevitably in this case the RCT will have to be repeated on a larger scale which is wasteful of resources and may be considered unethical because overall more cattle will have to be randomised to receive inferior treatments.

Within the framework of Bayesian statistics, a RCT can be formally designed to take into account the pre-existing beliefs of practitioners, which inevitably exist, such that if the trial finds a positive effect it is at least likely to be taken seriously by practitioners, given their current beliefs and may also avoid the costs associated with repeating the trial.

The focus of this research was to numerically capture the clinical beliefs of practising veterinary surgeons regarding the efficacy of systemic antibiotics when used in combination with intra-mammary dry cow therapy. The most common reason for prescribing long-acting antibiotic products to adult UK dairy cattle is for the purpose of dry cow therapy and several licensed intra-mammary dry cow therapy (IDCT) products exist. Systemic antibiotics may be administered as an adjunct to IDCT and this is currently common clinical practice in the UK. However systemic antibiotics are not licensed for dry cow therapy; for this purpose they can only be administered under the prescribing cascade at the discretion of individual veterinarians, who must justify the “off-licence” use. There is a recognised lack of robust data regarding this off-licence use, despite the fact that the responsible use of antibiotics in medicine is crucial, with bacterial resistance an ever-increasing concern.

A statistical technique called probabilistic elicitation was used to capture the veterinarians’ beliefs as probability distributions (often termed “prior beliefs” or simply “priors”). Probabilistic elicitation has been applied in a wide variety of fields and an extensive literature exists (O’Hagan et al., 2006). Once clinical beliefs have been numerically quantified as probability distributions two investigations can be carried out. Firstly, the diversity and strength of beliefs amongst the population can be studied. Secondly, the practitioners’ beliefs can be used within a Bayesian statistical framework to assess the strength of future evidence needed to *theoretically* convince the majority of practitioners interviewed that systemic antibiotics *do not* offer a clinically worthwhile benefit over IDCT alone. This aim stemmed from the fact that despite the current common use of systemic antibiotics for dry cow therapy, there is a lack of solid evidence supporting its efficacy and it is not an unreasonable proposition that such use may not be clinically worthwhile. Moreover systemic antibiotics in this context are off-licence and may contribute to antimicrobial resistance.

The two key aims of this research were therefore: (i) to conduct a probabilistic elicitation to quantify the variation in veterinarians’ beliefs regarding the efficacy of systemic antibiotics as an adjunct to IDCT and (ii) to investigate the strength of future research evidence that would be required (in theory) to change the beliefs of practising veterinary surgeons regarding the efficacy of systemic antibiotics, given their current clinical beliefs.

## 2 Materials and methods

The elicitation process can be considered to have 3 distinct phases each of which are equally important (Garthwaite et al., 2005; O’Hagan et al., 2006): (i) preparation (including identification and recruitment of experts, definition of variables, task structure), (ii) the elicitation itself, and (iii) further analysis dependent on the purpose of the elicitation and including an assessment of phases (i) and (ii). Each phase is described in turn.

### 2.1 Identification and recruitment of veterinarians

The target population comprised all veterinarians regularly involved in treating dairy cattle in England. The study population was the subset of veterinarians working within travelling distance of the first author's location; an area 100 miles in radius and centred on the University of Nottingham. The online database (http://www.rcvs.org.uk/) supplied by the Royal College of Veterinary Surgeons (R.C.V.S) provided a searchable sampling frame of veterinary practices. Only veterinary practices that treated cattle and contained at least 1 veterinarian holding the R.C.V.S post-graduate Certificate in Cattle Health and Production (“CertCHP”) were selected. Within the study area this yielded 13 veterinary practices (labelled 1–13 for anonymity) containing 77 practitioners treating dairy cattle in total.

Practitioners were paid at a rate of £100 per hour (pro-rata) for their time in order to encourage participation and minimise the non-response rate. It was desirable to use an equal probability selection method (“epsem” sample) with individual veterinarians having approximately the same probability (1/77) of selection (Barnett, 1974). A 2-stage cluster design was used where, in this context, a cluster refers to a single veterinary practice and these two terms are hereafter used interchangeably. Clusters were selected first with probability proportional to their size (i.e. the number of veterinarians they contained that treated dairy cattle) and then 5 veterinarians were selected randomly from within the chosen clusters. Thus larger clusters were more likely to be selected than smaller clusters but, if chosen, individuals within larger clusters then had less chance of selection compared to individuals within smaller clusters. This ensured that the probability of any individual veterinarian being selected was the same irrespective of the size of the cluster they worked in. A without-replacement systematic method was used to avoid the same cluster being selected twice, as follows (Kalton, 1983). Each cluster was assigned as many numbers as its size, such that cluster 1 (7 veterinarians) was assigned numbers 0–6, cluster 2 (8 veterinarians) numbers 7–14 and so forth. Dividing total size (77) by 5 (the pre-determined number of clusters to be selected) gave a sampling interval of 15.4. The random number generator function in the software programme “R” version 2.10.1 (R Development Core Team, 2009) was used to select a number between 0 and 15.4, say 9, to choose the first cluster (here cluster 2). Note that the number “9” was only used to select a cluster with probability proportional to cluster size and not to select individual veterinarians. Thereafter, 15.4 was added to 9 to give 24.4 (rounded down to 24) to deterministically select the next cluster and so forth. This method will only ensure that the same cluster is not selected twice if the sampling interval is greater than the largest cluster (as was the case here, 15.4 > 12). Once clusters were selected, named individuals within them were assigned numeric identifiers and 5 of these identifiers were selected randomly using the random number function generator in R. Since 3 clusters contained less than 5 veterinarians, all veterinarians in these clusters were guaranteed to be selected in the event of their cluster being chosen. This sampling design produced a sample of 24 veterinary surgeons (not 25 because 1 cluster selected only contained 4 veterinary surgeons) which met the financial/practical constraints placed on the study. Data were collected on 5 separate days over a 3-week period from 23rd June to 7th July 2010. Two pilot interviews with clinical academics from the University of Nottingham were conducted prior to main data collection to test that the method was tenable.

### 2.2 Definition of variables and task structure

The question of interest was “do systemic antibiotics in combination with IDCT offer a clinically worthwhile benefit over IDCT alone”? Thus it concerned a contrast between 2 unknown variables; the cure rate achievable with treatment 1 (denoted *θ*_1_) and the cure rate achievable with treatment 2 (denoted *θ*_2_) where “treatment 1” refers to IDCT alone and “treatment 2” refers to systemic antibiotics in combination with IDCT. The variables, *θ*_1_ and *θ*_2_, are probably regarded as dependent by the majority of veterinarians; given knowledge that *θ*_1_ is lower than expected, many veterinarians may believe (and it is biologically possible) that *θ*_2_ will also be lower. To quantify beliefs concerning 2 variables in full requires their joint probability distribution and in the case of dependent variables the task of elicitation becomes considerably more complicated (O’Hagan et al., 2006). To avoid this complexity it is sometimes possible to re-structure the problem so that is it expressed in terms of independent variables; the joint distribution is then just the product of the marginal densities and the task simplifies to eliciting beliefs about the marginal distribution for each variable separately. This elicitation task was re-structured by defining an additional variable, *θ*_3_, the cure rate achievable with treatment 2 given treatment 1 has failed. With this definition for *θ*_3_, *θ*_2_ is given by

(1) $$\theta_{2}=\theta_{1}+(1-\theta_{1})\theta_{3}\text{.}$$

Thus *θ*_1_ and *θ*_3_ were elicited separately to obtain the marginal distributions of each; denoted *f*(*θ*_1_) and *f*(*θ*_3_). Since 0 ≤ *θ*_1_ ≤ 1 and 0 ≤ *θ*_3_ ≤ 1, parametric distributions from the beta family were fitted to the elicited values for *θ*_1_ and *θ*_3_ since this family has flexibility to cover a wide range of beliefs and avoids any issues with impossible events. Hence

$$\begin{array}{c} (\theta_{1})∼Beta(\alpha ,\beta );\;f(\theta_{1})=\frac{1}{B(\alpha ,\beta )}\theta_{1}^{\alpha -1}{\left(1-\theta_{1}\right)}^{\beta -1}\;\text{and}\\ \theta_{3}∼Beta(\alpha^{\prime },\beta^{\prime });\;f(\theta_{3})=\frac{1}{B(\alpha^{\prime },\beta^{\prime })}\theta_{3}^{\alpha^{\prime }-1}{\left(1-\theta_{3}\right)}^{\beta^{\prime }-1}, \end{array}$$

where $B(\alpha ,\beta )=\int_{0}^{1}\theta^{\alpha -1}{\left(1-\theta \right)}^{\beta -1}d\theta$ and *α* > 0, *β* > 0 are the hyperparameters for the marginal distribution of *θ*_1_and similarly *α*′ > 0, *β*′ > 0 for *f*(*θ*_3_). The assumption of independence between *θ*_1_ and *θ*_3_ was considered acceptable to veterinary surgeons and hence the joint distribution of *θ*_1_,*θ*_3_ is given by *f*(*θ*_1_,*θ*_3_) = *f*(*θ*_1_)*f*(*θ*_3_) Since T: (*θ*_1_,*θ*_3_) → (*θ*_1_,*θ*_2_) is a one-to-one transformation of continuous variables, the joint distribution of *θ*_1_ and *θ*_2_ involved a standard calculation using the Jacobian to give

(2) $$f(\theta_{1},\theta_{2})=\left\{\begin{array}{cc} \phi \theta_{1}^{\alpha -1}{\left(1-\theta_{1}\right)}^{\beta -2}{\left(\frac{\theta_{2}-\theta_{1}}{1-\theta_{1}}\right)}^{\alpha^{\prime }-1}{\left(\frac{1-\theta_{2}}{1-\theta_{1}}\right)}^{\beta^{\prime }-1} & \text{if}\;0\leq \theta_{1}\leq \theta_{2}\leq 1,\\ 0 & \text{otherwise,} \end{array}\right\}$$

where *ϕ* is a normalising constant, *ϕ* = (1/*B*(*α*, *β*))(1/*B*(*α*′, *β*′)). However structuring the problem according to Eq. (1) also necessitates that *θ*_2_ is greater than or equal to *θ*_1_. It raised the question of whether this would impose an unrealistic constraint on the veterinarians’ beliefs. However whilst it is biologically possible that *θ*_2_ < *θ*_1_ (i.e. that less cows would be cured by administering systemic antibiotics in addition to IDCT) the authors believed that the majority (if not all) veterinarians would take the view that at worst *θ*_2_ = *θ*_1_. Nevertheless, the belief that *θ*_2_ < *θ*_1_ was still elicited separately to capture this opinion, should it exist.

### 2.3 Probabilistic elicitation method

The technique is an iterative process that usually involves eliciting a small number of summaries of the expert's belief, fitting parametric distributions to them and assessing the adequacy of the fit (Garthwaite et al., 2005). The Sheffield Elicitation Framework (SHELF, O’Hagan and Oakley; http://www.tonyohagan.co.uk/shelf/) was used in this study; it is a freely available package of guidance documents, templates and software specifically designed for carrying out probabilistic elicitation. Current best practice for probabilistic elicitation was employed whereby an interview between the first author and each practitioner was conducted and feedback carried out using SHELF software, version 1.01. This involved presenting the fitted distribution to the veterinarian graphically and describing some of the implied (but not elicited) probabilities. The veterinarian then had the opportunity to disagree with any implied assertions which were revised until the fitted distribution was considered a fair reflection of their beliefs. This is essential because it is impossible to directly judge if any elicitation has been “successful”; apart from elicitation itself, there is no other method to establish what the person actually believes (Garthwaite et al., 2005).

For each variable (*θ*_1,_*θ*_3_), 5 values were elicited to allow a distribution to be fitted: the plausible range (minimum to maximum), median and 2 further probability judgements (lower and upper quartiles). Hence the total number of observations per veterinarian was 10. The R code provided in SHELF was used to fit beta distributions to *θ*_1_ and *θ*_3_. This uses numerical optimisation based on the simplex algorithm (Nelder and Mead, 1965) to select the best fitting hyperparameters by minimising the sum of the squared differences between the fitted cumulative distribution and the elicited cumulative distribution.

For the elicitation itself, veterinarians were asked to consider commercial dairy cows at the point of drying-off which had a chronic intra-mammary infection (in 1 or more quarters) with unknown but major pathogens; “chronic” was defined as a somatic cell count greater than 400,000 cells per ml at both of the 2 monthly milk recordings prior to drying off. Cows received either treatment 1 or treatment 2 and it was assumed that no other treatments were given until calving. Cows were cured if they were free from any major intra-mammary pathogens at calving. Note that the actual product choice for both treatment 1 and 2 was left to the discretion of the veterinarian; interest resided in the generic use of antibiotics administered by the systemic route for dry cow therapy. However it was stipulated that the choices must be products the veterinarians would actually prescribe in practice, with the objective of optimising cure rates.

A standard script was used for consistency (available online as a supplementary file; Appendix A). The task was presented as a probabilistic judgement in relative frequency terms for simplicity. Hence the question was based on the number of cows (out of 100) that it was believed could be cured with each treatment and the associated uncertainty around this number. However within this context it was important to avoid any possible confusion between *epistemic* uncertainty, originating from imperfect knowledge, and *aleatory* uncertainty arising due to randomness (O’Hagan et al., 2006). Of interest was an epistemic variable, the true cure rate achievable with each treatment. Aleatory uncertainty due to the context of considering only a theoretical sample of 100 cows was not of interest and it was undesirable for veterinarians to include this extra uncertainty in their assessments; the standard script contained a statement to clarify this.

The elicitation was also designed to avoid heuristics, which are mental strategies people use to make numerical assessments in the face of uncertainty; they can be effective but may lead to severe systematic bias and error. A large number of heuristics have been identified (Cooke, 1991; Garthwaite et al., 2005; O’Hagan et al., 2006). In particular, “anchoring-adjustment” heuristics were avoided by deliberately not providing initial values or describing a specific clinical scenario. This type of heuristic is also important with respect to the order in which elicited values are requested; experts were asked for their plausible range first so that further judgements were made relative to this. There is also substantial evidence to suggest that people do not assign enough probability to the tails of their distribution (Alpert and Raiffa, 1982; Garthwaite et al., 2005); careful phrasing of questions about the plausible range is required. For example, the minimum value was established by asking “tell me the least number of cows you believe will be cured such that you think it is extremely unlikely (but not impossible) that less than this number would be cured”.

Information was gathered during the interview concerning key features of both the individual veterinary surgeons and the veterinary practices they worked in. The raw data were entered into Microsoft Excel (Version 2007, Microsoft Corp). All subsequent data analysis was carried out within the R programming environment, version 2.10.1 (R Development Core Team, 2009).

### 2.4 Classical multidimensional scaling

For this analysis the elicited values from the 2 pilot interviews were included taking the total number of veterinarians from 24 to 26. If the vector of 10 elicited values for each veterinarian is denoted ***x***_*i*_ (*i* = 1, …, 26) then the squared Euclidean distance between the vectors for veterinarian *i* and veterinarian *j* is given by (***x***_*i*_ − ***x***_*j*_)^*T*^(***x***_*i*_ − ***x***_*j*_) and this was used as a measure of the dissimilarity in veterinary beliefs. The 26 by 26 “distance” matrix, ***D*****,** used to classically scale the data was given by

$$D=[d_{ij}]=\sqrt{{\left(x_{i}-x_{j}\right)}^{T}(x_{i}-x_{j})}\text{,}$$

where *i*, *j* = 1, …, 26. The standard “goodness of fit” statistic was calculated; it describes the proportion of the total variation accounted for by the first *m* dimensions and is given by $\sum_{j=1}^{m}\lambda_{j}/\sum_{i=1}^{10}\lambda_{i}$ where *λ*_*j*_ are the eigenvalues of the centred inner product matrix, obtained by centring the squared distance matrix, ***D***, times −1/2 (Mardia et al., 1979; Cox and Cox, 2000). This was used to determine the appropriate number of dimensions in conjunction with a scree plot. Interpretation of the principal coordinate axes was facilitated by inspection of the associated eigenvectors.

### 2.5 Bayesian analysis

Bayes’ theorem mathematically describes the rational way to update an initial state of knowledge about an unknown variable to a new state of knowledge in the light of new data. For a single continuous unknown variable *θ*, Bayes’ theorem can be written as

(3) π(θ|x)∝π(x|θ)π(θ),

where *π*(*θ*) is the prior probability density function (or prior belief), *π*(***x***|*θ*) is the likelihood (based on new data ***x***) and *π*(*θ*|***x***) is the posterior probability density function containing all available information about *θ* after the prior belief has been updated with information contained in the data. Eq. (3) states that the extent of any logical change in belief depends on both the prior belief, *π*(*θ*), and the strength of the new evidence, *π*(***x***|*θ*).

It is important to note that during their interviews veterinarians were simply asked for their current clinical beliefs, *π*(*θ*), and they were not shown any hypothetical data. Instead, the Bayesian analyses were performed entirely using statistical models (after the interviews had been conducted), whereby the 24 priors obtained from each veterinarian were combined (individually) using Bayes’ theorem with synthetic data from clinical trials (of different sizes) for the likelihood, *π*(***x***|*θ*), in order to produce 24 posterior distributions, *π*(*θ*|***x***). The posterior distributions can be considered to represent what each veterinarian would believe if they were shown the synthetic data and they updated their prior beliefs rationally in accordance with Bayes’ theorem. The posterior distributions are therefore referred to subsequently in this manuscript as “predicted beliefs” (one associated with each veterinarian).

For this analysis a threshold was used; a “clinically worthwhile benefit” was taken to be a minimum of a 5–10% improvement in the probability of cure with treatment 2 over treatment 1 (equivalent to an odds ratio ≥ 1.5).

The synthetic clinical trial data comprised *n*_1_ infected cows randomly assigned to treatment 1 and *n*_2_ infected cows to treatment 2, with *n*_1_ = *n*_2_. The outcome is binary; an infected cow either cures or does not cure. Cows receiving treatment 1 are cured with probability *θ*_1_ and cows receiving treatment 2 are cured with probability *θ*_2_. If a trial was actually conducted in reality, then the observed data would be realisations on random variables *X*_1_ (the number of cows cured who received treatment 1) and *X*_2_ (the number of cows cured who received treatment 2), and we are told that *X*_2_ ≥ *X*_1_. From these observed data, information concerning *n*_3_, the number of cows not cured by treatment 1, can be calculated from *n*_3_ = *n*_1_−*X*_1_ and information regarding random variable *X*_3_ (the extra number of cows cured by treatment 2 compared to treatment 1) can be calculated from *X*_3_ = *X*_2_ − *X*_1_. For this Bayesian analysis, *X*_1_was assumed to follow a binomial distribution, *X*_1_ ∼ *Binomial* (*n*_1_, *θ*_1_). The conditional distribution of *X*_3_ given *X*_1_was also assumed to follow a binomial distribution, *X*_3_|*X*_1_ ∼ *Binomial* (*n*_3_, *θ*_3_) where *θ*_3_ is the probability of cure with treatment 2, given treatment 1 has failed. Prior marginal distributions for independent variables *θ*_1_ and *θ*_3_ had been elicited for each veterinarian (Section 2.3). A prior distribution was not elicited directly for *θ*_2_ due the complication that arises due to its dependency on *θ*_1_ (Section 2.2). Synthetic data were generated for different sized trials: 30, 50, 250, 500, 750 and 1000 infected cows in each treatment arm. In each case, *θ*_1_ and *θ*_2_ were set equal so that *θ*_3_ = 0, i.e. the synthetic data suggested there was no difference between the 2 treatments. The Bayesian model was

$$\begin{array}{c} \theta_{1i}∼Beta(\alpha_{i},\beta_{i}):\;\text{prior belief for}\;\theta_{1}\;\text{for the}\;i\text{th veterinarian},\\ \theta_{3i}∼Beta({\alpha^{\prime }}_{i},{\beta^{\prime }}_{i}):\;\text{prior belief for}\;\theta_{3}\;\text{for the}\;i\text{th veterinarian}, \end{array}$$

where *α*_*i*_, *β*_*i*_ and ${\alpha^{\prime }}_{i},{\beta^{\prime }}_{1}$ are the fitted hyperparameters for the *i*th veterinarian (*i* = 1, …, 24) and the likelihood was

$$L(X_{1},X_{3}|\theta_{1},\theta_{3})=\left(\begin{array}{c} n_{1}-x_{1}\\ x_{3} \end{array}\right)\theta_{3}^{x_{3}}{\left(1-\theta_{3}\right)}^{n_{3}-x_{3}}\left(\begin{array}{c} n_{1}\\ x_{1} \end{array}\right)\theta_{1}^{x_{1}}{\left(1-\theta_{1}\right)}^{n_{1}-x_{1}}\text{.}$$

This model is a conjugate analysis and using Bayes’ theorem, Eq. (3), the joint posterior distribution for *θ*_1_, *θ*_3_ given the data is

$$\pi (\theta_{1},\theta_{3}|X_{1},X_{3})\propto \theta_{1}^{x_{1}+\alpha_{i}-1}{\left(1-\theta_{1}\right)}^{n_{1}-x_{1}+\beta_{i}-1}\theta_{3}^{x_{3}+{\alpha^{\prime }}_{i}-1}{\left(1-\theta_{3}\right)}^{n_{3}-x_{3}+{\beta^{\prime }}_{i}-1}\text{,}$$

from which it can be seen that the marginal posterior distributions of *θ*_1_ and *θ*_3_ given the data follow independent beta distributions

$$\begin{array}{c} \theta_{1}|X_{1}X_{3}∼Beta(\alpha_{i}+x_{1},\beta_{i}+n_{1}-x_{1}),\\ \theta_{3}|X_{1}X_{3}∼Beta({\alpha^{\prime }}_{i}+x_{3},{\beta^{\prime }}_{i}+n_{3}-x_{3}). \end{array}$$

However, the posterior distribution of primary interest was the odds ratio of *θ*_2_ to *θ*_1_, (*θ*_2_/1 − *θ*_*2*_)/(*θ*_1_/1 − *θ*_1_) for each veterinarian, given the data. Therefore simulated values from the joint posterior distribution of *θ*_1_*θ*_3_|*X*_1_*X*_3_ where transformed directly by calculation using Eq. (1), to give simulated values for the joint posterior distribution, *θ*_1_*θ*_2_|*X*_1_*X*_3_ and this in turn was used to obtain the marginal posterior distribution for the odds ratio of *θ*_2_ to *θ*_1_, given the data.

Software developed by the “BUGS” project (Bayesian inference Using Gibbs Sampling; http://www.mrc-bsu.cam.ac.uk/bugs/welcome.shtml) was used in a form embedded within R, the library “BRugs” (Thomas et al., 2006) to run the model which uses Markov chain Monte Carlo (MCMC) stochastic simulation.

### 2.6 Sensitivity analysis

Sensitivity analysis is important to investigate any imprecision in the priors introduced by fitting parametric beta distributions to a small number of elicited summary statistics (Garthwaite et al., 2005). It is desirable that the analysis is robust to realistic alternative choices of family, in the sense that the veterinarians would be likely to recognise the fitted distributions as a reflection of their own beliefs. Hence alternative choices for the parametric distributions (including the normal and gamma families) were explored and the analysis re-run to investigate the consequences for different sizes of clinical trial.

## 3 Results

### 3.1 Descriptive statistics

**Fig. 1:**
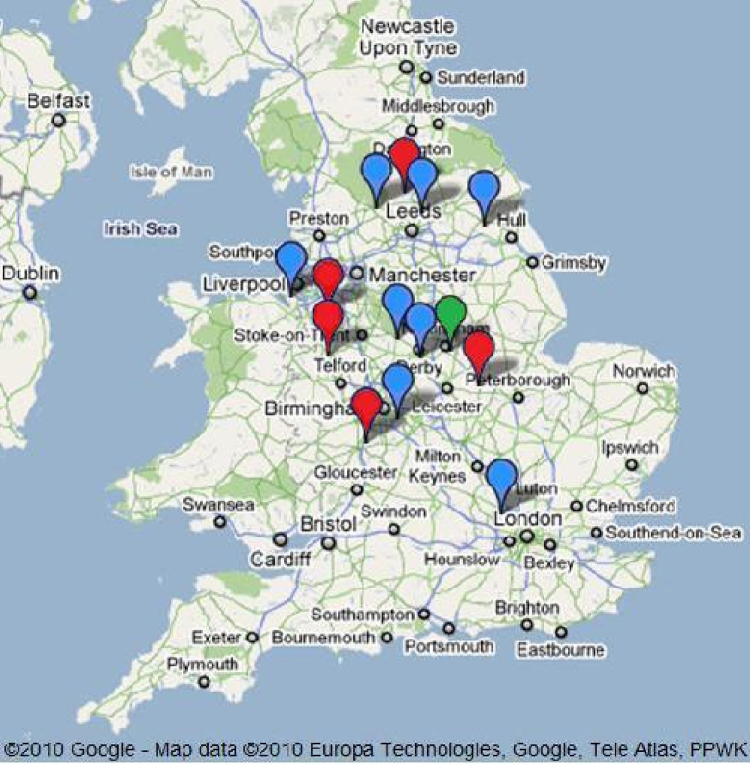
Map of England showing all 13 veterinary practices (clusters) within the sample area; clusters visited are marked in black (red, colour version). Nottingham is marked in light grey (green, colour version).

In total 24 veterinarians from 5 practices were interviewed for up to 40 min each; 4 veterinarians from 1 practice and 5 from each of the other 4 practices. Fig. 1 shows the location of the practices in the sample area and those visited. The non-response rate was zero. In terms of type of species treated by the practice, 2 were “mixed species” and the remaining 3 were “farm and equine only”, “farm only” and “dairy only”, respectively. The veterinarians within the clusters varied widely with respect to several important characteristics likely to influence clinical beliefs. Seven of the veterinarians held extra cattle-related qualifications; 5 held the CertCHP, 2 held the Diploma in Bovine Reproduction and 1 held both. The number of years qualified varied from 9 months to 26 years (median 7 years). The “percentage of current time spent working with dairy cattle” ranged from 15–100% (median 80%). The “percentage of that time dedicated to dairy preventive medicine work” varied from 0% to 50% (median 16%). Number of “dairy clients primarily responsible for” ranged from 0 to 35 (median 10.5). Number of “days dedicated to dairy cattle specific continuing professional development in the last 12 months” varied from 0–20 (median 4). Gender was split 20 males to 4 females. There were 11 assistants and 13 partners; all worked full-time. All 6 UK veterinary schools were represented at least twice. Liverpool graduates dominated (46% of the sample) possibly reflecting this school's geographic proximity to the study population. Two veterinarians qualified abroad. The sample appeared to be a fair representation of the different types of veterinarians and veterinary practices that treat dairy cattle.

**Fig. 2:**
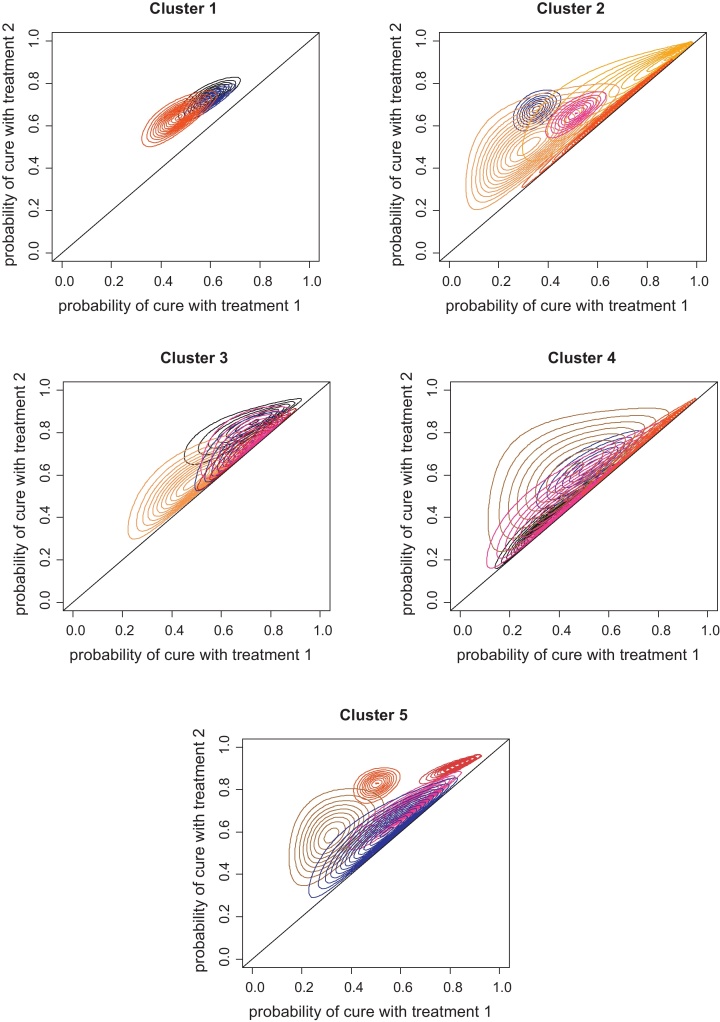
Joint probability distributions (*θ*_1_, *θ*_2_) for each individual veterinarian displayed as contour plots and grouped by veterinary practice (cluster). Treatment 1 = intra-mammary dry cow therapy (IDCT) alone. Treatment 2 = systemic antibiotics plus IDCT. *θ*_1_ = the probability of cure with treatment 1 and refers to commercial dairy cows at calving, who at the point of drying-off received treatment 1 and had chronic (somatic cell count ≥400,000 cells per ml) intra-mammary infections (≥1 quarters) due to (unknown) major pathogens; and similarly, *θ*_2_ = the probability of cure with treatment 2. The black diagonal line denotes *θ*_1_ = *θ*_2_.

When asked for the probability that *θ*_2_ < *θ*_1_ (i.e. that the additional use of systemic antibiotics would result in less cows cured) 15 of the 24 veterinarians stated *P*(*θ*_2_ < *θ*_1_) = 0 and the remainder gave *P*(*θ*_2_ < *θ*_1_) ≤ 0.05. Hence structuring the task according to Eq. (1) was considered justifiable and Eq. (2) was used to infer the joint distribution *f*(*θ*_1_,*θ*_2_) for each veterinarian. Fig. 2 displays the joint distributions as contour plots and illustrates the diversity in clinical beliefs; there are striking differences both within and between practices with respect to central location and dispersion of beliefs. Both “confident optimism” and “confident pessimism” for the combined use of systemic and intra-mammary antibiotics are observed, alongside several veterinarians who had considerable uncertainty.

### 3.2 Classical multidimensional scaling

The first 3 (of 10 possible) dimensions accounted for 95.3% of the variation and were sufficient to portray the data structure. The first principal coordinate axis was interpreted as an overall measure of the veterinarians’ belief for *θ*_3_. Hence it contained information related to all 5 elicited judgements for this variable and therefore incorporated not just the belief regarding the median, but also the uncertainty (minimum and maximum cure rates achievable) and an impression of the shape of the elicited distribution. Similarly, the second principal coordinate axis was interpreted as an overall measure of the veterinarians’ belief for *θ*_1_. The third principal coordinate axis was a contrast between the elicited minimum and maximum cure rates for *θ*_3_. It reflected the veterinarians uncertainty alone in *θ*_3_.

**Fig. 3:**
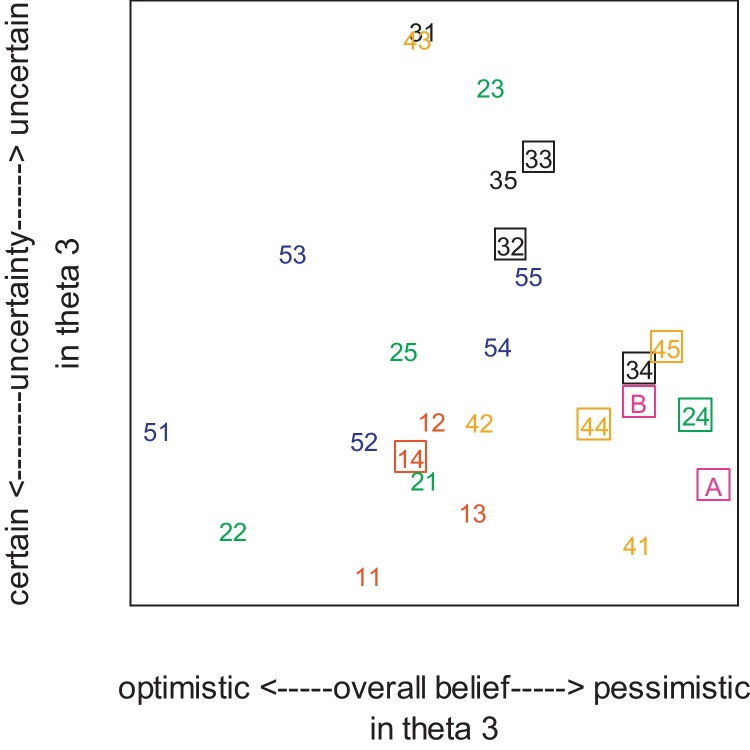
Classical scaling of the data. Each number represents an individual veterinarian, with those from the same practice sharing the same first digit (e.g. 11, 12, 13, 14 all worked for the same practice, labelled “1”). A square box highlights those holding extra qualifications. “A” and “B” represent the clinical academics from the University of Nottingham. Theta 3 = *θ*_3_ = the probability of cure with treatment 2, given treatment 1 has failed.

The two-dimensional map for one of the three planes is presented in Fig. 3. Each number represents an individual veterinarian, with those from the same practice sharing the same first digit (e.g. 11, 12, 13, 14 all worked for the same practice, labelled “1”). A square box highlights veterinarians holding extra qualifications (denoted “expert”). “A” and “B” represent the clinical academics from the University of Nottingham. Fig. 3 reveals that 6 of the 9 expert veterinarians (clustered in the lower right corner of the map) shared the same “confident pessimism” for *θ*_3_ and whilst 2 experts (32 and 33 from the same practice) were slightly more optimistic, they were also much more uncertain. Only 1 expert (14) had “confident faith” in *θ*_3_. Inspection of the other 2 planes (not shown) revealed that 7 of the experts shared their optimism for *θ*_1_ (the success of IDCT alone), whilst the other 2 experts (32, 14) were more pessimistic.

Two notable outliers (in all 3 planes) were 41 (representing “confident pessimism”) and 51 (“confident optimism”) about both *θ*_1_and *θ*_3_. It can be seen from both Figs. 2 and 3 that the veterinarians in practice 1 are located closest together, suggesting that they held the most similar beliefs (and with a similar level of “firm confidence”) of any practice. In particular, the raw data showed that they all believed systemic antibiotics would cure a minimum of 20% more cows (given IDCT had failed). In comparison, the veterinarians in practice 5 showed a much greater diversity of opinion and strength of belief (Figs. 2 and 3); yet nevertheless, the raw data revealed that all the veterinarians in practice 5 did agree that systemic antibiotics would have at least some benefit. In contrast, the raw data showed that everyone in practice 3 agreed that using systemic antibiotics may not improve cure rates at all; a view also shared by 4 of the 5 veterinarians in practice 4.

### 3.3 Bayesian analysis

**Fig. 4:**
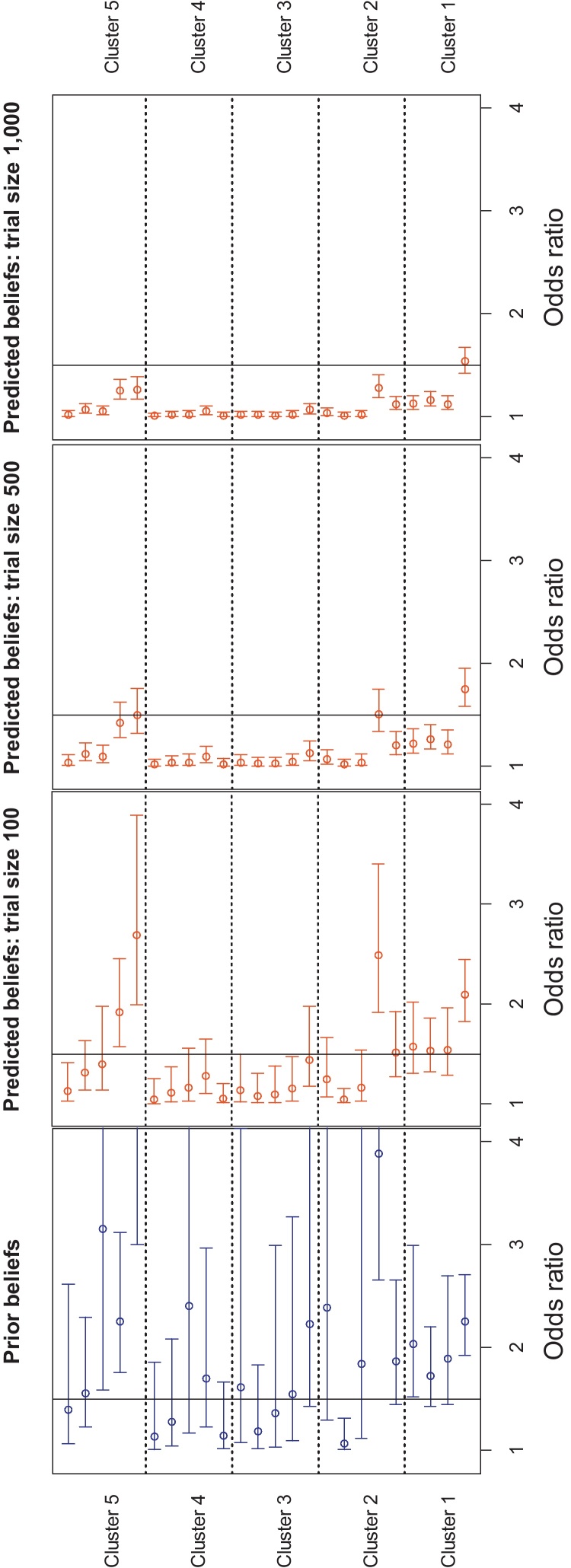
The prior beliefs (column 1) and posterior beliefs (columns 2–4) 95% credible intervals for the odds ratio for the 24 veterinarians. The likelihood was based on synthetic data from a single clinical trial that showed no difference between treatment 1 and 2 and total trial size = 100, 500 and 1000 infected cows. An odds ratio ≥ 1.5 was the threshold used for a “clinically worthwhile benefit” with treatment 2 compared to treatment 1 (i.e. the adjunct use of systemic antibiotics for dry cow therapy). *Note*: The choice of *x*-axis scale truncates 8 of the prior credible intervals at their upper range, but this choice facilitates comparison with the posterior predicted beliefs.

MCMC simulation was used with 3 chains, a total sample size of 30,000 and a “burn-in” of 1000 iterations. The chains visually converged almost immediately; Gelman–Rubin statistic convergence to 1. Fig. 4 (first column) shows the 95% credible intervals for the prior distributions of the odds ratio for the 24 veterinarians (note: the choice of *x*-axis scale truncates 8 of the prior credible intervals at their upper range, but this choice facilitates comparison with the posterior predicted beliefs). Only 1 veterinarian had their entire 95% prior credible odds ratio interval below 1.5, whilst 6 veterinarians had their entire 95% intervals above 1.5 (recall that an odds ratio ≥ 1.5 was taken to indicate that systemic antibiotics do provide a clinically worthwhile adjunct to intra-mammary dry cow therapy). Fig. 4 (second to fourth columns) shows the 24 posterior distributions (i.e. the “predicted beliefs”) derived from combining the priors from the 24 veterinarians with synthetic data from clinical trials of varying size (50, 250 and 500 cows in each treatment group), each of which shows no difference between treatments. Fig. 4 reveals that only with a trial size of 1000 cows (500 in each treatment arm) are 23 of the 24 predicted beliefs such that the entire 95% posterior credible odds ratio intervals are less than 1.5. A trial size of 2000 would be required for the 95% credible intervals of all 24 predicted beliefs to be less than 1.5. From Fig. 4 it can also been seen that the uncertainty in the prior is very important with respect to updating from prior to posterior distributions using Bayes’ theorem, more so than the centre of location of the prior.

### 3.4 Sensitivity analysis

For realistic alternative distributions the results were very similar to the original analysis; the number of predicted beliefs crossing the threshold in their entirety at each trial size altered by at most 2. Hence any imprecision arising in the priors associated with fitting parametric distributions was not a primary concern.

## 4 Discussion

This study has demonstrated how probabilistic elicitation can be used to capture the diversity and strength of veterinarians’ beliefs for subsequent use as prior information in a Bayesian analysis. Here we have used synthetic data from clinical trials of varying sizes and Bayes’ theorem to illustrate how the clinical beliefs of these 24 practitioners should (rationally) be updated in the light of new evidence. This may be described as a true Bayesian approach in the sense that genuine prior knowledge has been utilised in a scientific and transparent way; it differs to the majority of published Bayesian analyses that use a variety of theoretical prior beliefs (O’Hagan, 2006).

A major advantage of adopting a true Bayesian approach is that by eliciting clinicians’ beliefs at the design stage of clinical trials, sample size calculations are facilitated by placing the proposed trial in the context of current clinical opinion. Despite increasing use of this approach in human medicine over the last decade (Spiegelhalter et al., 2004), the authors are not currently aware of any veterinary research trials that have been designed in this way. Yet research efforts will obviously be targeted more effectively if trials destined not to sway clinical beliefs from the very outset are avoided. This is particularly important where there are strongly held and/or diverse pre-existing clinical beliefs amongst veterinarians; and it is postulated that this may frequently prove to be the case in farm animal veterinary medicine. In analogy with human medicine, an important reason why veterinary research may have failed in the past to evoke change could be due to the strength of the research produced relative to the strength and diversity of practitioners’ existing beliefs. We would urge more consideration of this issue.

Possible reasons why probabilistic elicitation has not been used more widely in the veterinary field include the complexity of the task, cost and time considerations (Berry and Stangl, 1996). Indeed, as Spiegelhalter et al. (2004) commented “turning informally expressed opinions into a mathematical prior distribution is perhaps the most difficult aspect of Bayesian analysis”. However it is hoped that the development of freely available software (such as SHELF) along with the major benefits to be derived from conducting productive research, will provide motivation. In particular, it is essential that veterinary research which has implications for wider society (e.g. in terms of public health, or informing government animal health policy on a national scale) is designed with due respect for the pre-existing beliefs of all relevant stakeholders.

Designing a clinical trial to investigate the adjunct use of systemic antibiotics for dry cow therapy that is based on a null hypothesis of treatment equivalence in cure rates is unrealistic; treatment 2 (systemic therapy in addition to IDCT) will always carry an increased cost over treatment 1 (IDCT alone) and other possible differences between the treatments (such as side effects, toxicity and drug resistance) mean that a certain improvement in cure rate with treatment 2 will be required by veterinarians before it would be considered clinically superior to treatment 1. Hence in this paper a “clinically worthwhile benefit” was used instead and assumed by the authors to be at least a 5–10% improvement in the cure rate with treatment 2 compared to treatment 1 (odds ratio ≥ 1.5). However it is worth noting that elicitation of clinicians’ beliefs concerning a “clinically worthwhile benefit” has been carried out in human medicine at the same time as eliciting clinicians’ prior expectations regarding the treatment effect (Parmar et al., 1994) and can be used to inform the choice of null hypothesis. Careful distinction between clinical *demands* and clinical *expectations* is crucial to such an elicitation task, particularly when these are quantitatively similar (Spiegelhalter et al., 2004).

This paper describes the use of a clinical trial for making inferences (i.e. to update an existing state of knowledge (clinical belief) to a new state of knowledge) and does not go beyond that to formally consider clinical decision making. Decision making involves the integration of clinical beliefs (based on a summary of all available evidence and associated uncertainties) with an assessment of all relevant utilities (also termed “values”) and is dependent on context; Bayesian statistical decision theory provides a natural framework for the formal assessment of such evidence-based clinical decision making (Ashby and Smith, 2000).

There were major variations in veterinarians’ beliefs with respect to the efficacy of systemic antibiotics for dry cow therapy, both between individuals within a practice and between practices. Striking differences in the strength of belief were also apparent. Although decision making has not been formally considered here, nevertheless the wide diversity in clinical beliefs observed is likely to result in very different decisions being taken on farm, with considerable discrepancies in the treatments received by dairy cows at drying-off and the total quantity of systemic antibiotics being administered. The observed variation raises considerable concern over whether or not antibiotics are being prescribed consistently and appropriately; any widespread misuse (or overuse) of antibiotics has serious implications (DEFRA, 2011). This research raises important questions about the heterogeneity in veterinarians’ beliefs in general. Whilst it would be undesirable for veterinarians to be completely unified in their clinical beliefs, broad agreement (particularly with respect to antibiotics) is important for both the credibility of the veterinary profession and to ensure a consistent delivery of healthcare to dairy cattle. Further research is certainly required in this area.

Explanation of the observed variation is likely to be multi-factorial and reasons for it could include: availability/cost of products and their marketing by pharmaceutical companies, differences in how veterinarians source and critically appraise information, the lack of robust data, under and post-graduate education, the creation and persistence of dogma, the absence of national guidelines, personality traits, farmer perceptions/demand and demographic factors.

Interestingly the majority of expert veterinarians in this sample shared a confidently pessimistic belief in the combined use of systemic and intra-mammary antibiotics, given intra-mammary antibiotics alone have failed, offering some support for the idea that post-graduate education is a factor related to the observed variation in beliefs. Moreover, whilst the beliefs of expert veterinarians do not constitute fact, they are nevertheless important, especially in the absence of robust data. Overall, when placed in the greater context of an escalating global threat of bacterial resistance to antibiotics, the justification for the off-licence use of systemic antibiotics for dry cow therapy becomes difficult.

Cluster sampling was used in this research and this method is only efficient when the clusters are internally as heterogeneous as possible with respect to survey variables and between cluster variation is small; the “clustering principle” (Stuart, 1983). Initial concern that the variables of interest (clinical beliefs) may be fairly homogenous within clusters (because veterinarians would discuss and share ideas amongst themselves) proved unfounded; as previously mentioned, striking differences in beliefs were observed both within and between clusters. Hence, for the purposes of eliciting veterinarians’ beliefs, cluster sampling may prove to be cost-efficient. The sample size in this study was 24 and one third (24/77) of the study population were interviewed. In a recent systematic review involving 33 published elicitations, Johnson et al. (2010) reported median sample size as 11, hence this elicitation was of relatively large scale in comparison with other studies currently published.

The R.C.V.S database was searched by selecting veterinary practices containing at least one veterinarian holding the “CertCHP”. It was believed that post-graduate qualifications may strongly influence clinical beliefs and it was of interest to include a number of expert veterinarians in the sample. This necessitated deliberate selection but accordingly, caution should be taken making any inferences to wider veterinary populations. The non-response rate was zero and thus veterinarian-induced selection bias was avoided; the relatively short interview time and payment for veterinary time may have influenced this appreciably.

## 5 Conclusions

This research has demonstrated the usefulness of probabilistic elicitation for evaluating the diversity and strength of practitioners’ beliefs. With respect to the efficacy of systemic antibiotics as an adjunct to ICDT, major variations in beliefs were observed both within and between practices and this raises concern over the use of these essential medicines. It is crucial to understand practitioners’ pre-existing beliefs when designing clinical trials. Further research to investigate the heterogeneity in beliefs of veterinarians is important.

## Conflict of interest

None declared.
