# Supplementary material for: A Bayesian elicitation of veterinary beliefs regarding systemic dry cow therapy: Variation and importance for clinical trial design
Source: Prev Vet Med. 2012 Sep 15;106(2):87–96. doi: 10.1016/j.prevetmed.2012.01.017 (PMC3437563; doi:10.1016/j.prevetmed.2012.01.017)
Supplement: Supplementary file 1 [file mmc1.doc]

Standard Script

Thank you for agreeing to meet with me and answer this questionnaire.

We are interested in capturing the beliefs of farm vets about the efficacy of intra-mammary dry-cow therapy (DCT) in comparison to systemic antibiotics plus DCT. The question we are asking is about something for which there is no “right or wrong” answer. We value your opinion because you have clinical experience. However, nobody can be expected to be “all-knowing” and it is natural that you will have some uncertainty associated with your answer. We are interested in capturing this uncertainty so that it represents what you believe to be true as accurately as possible.

We would also like to capture information about you as a veterinary surgeon so that we can identify factors that may be associated with different clinical opinions.

Finding out about clinical opinions, associated uncertainties and factors that influence them will help us to direct future research for the benefit of the profession.

This questionnaire is confidential and your details will be made anonymous and the results will only be used for research purposes.

Eligibility for project

Have you already discussed the content of this questionnaire with any other veterinary surgeons? (*yes/no*) If no, thank participant for their time. If yes, proceed.

It is *important* that you do not discuss this questionnaire afterwards with other vets in your practice or your neighbouring practices until after they have completed this questionnaire themselves.

Start (record time)

Your uncertainty about whether or not an event will happen can be expressed as a probability on a scale of 0 to1. Zero means the event can’t happen, one means that the event will definitely. A probability of 0.5 means you think there is a 50% chance the event will happen and a 50% chance it won’t. A probability of 0.8 means you think there is an 80% chance it will happen, and a 20% chance that it won’t.

Do you understand how to express your uncertainty as a probability? (*yes/no*) If yes, proceed. if no, clarify

Consider a group of 100 commercial dairy cows (of all different ages and from all different farms) which have a chronic intra-mammary infection (in one or more quarters) at the point of drying off with unknown major pathogens. (by “chronic” I mean that the somatic cell count has been over 400,000 cells/ml at both of the previous two monthly milk recordings)

All chronically infected cows receive the same treatment at drying-off. Assume that no other treatments are given until calving. We are interested in whether or not they are still infected at calving-in.

Do you understand so far? (*yes/no)* If yes, proceed. If no, clarify.

Consider that the 100 chronically infected cows all received:

*Long-acting intra-mammary dry cow therapy chosen by yourself*

Some of these 100 chronically infected cows may be “cured” and calve in *not* infected, but you cannot be expected to know exactly how many may have cured. It is natural that you will have some uncertainty.

Therefore can you give me a *range* for the number of cows that you think it is possible to cure (out of the 100 infected)? That is, tell me the *least* number (L) of cows you believe will be cured such that you think it is extremely unlikely that *less* than this number would cure and tell me the *maximum* number (U) of cows you would cure, such that you think it is extremely unlikely that *more* than this number could have cured. Note that in general, the *more* uncertain you are about the answer, the *wider* you range should be.

Also note that although we are only considering 100 cows, it is the *true* cure rate that we are interested in. If you are thinking about any uncertainty that may arise because this is only one of many possible samples of 100 cows, you should *ignore* this uncertainty and *not* include it in your assessment.

Do you have any questions? (*yes/no*) If yes, proceed. If no, clarify

Given your range, what is the median number (M) of cows that you might *typically expect* to cure? By “median” I mean you think there is a 50% chance that less than this average number will cure and a 50% chance that more than the average number will cure.

Given that you think that there is a 50% chance that between L and M will cure. What is the value X1 such you believe that the two intervals [L, X1] and [X1, M] are equally probable?

Given that you think that there is a 50% chance that between M and U will cure. What is the value X2 such you believe that the two intervals [L, X2] and [X2, M] are equally probable?

[Fit probability distribution, produce graph (using SHELF software). Show to vet (with explanation) and compare the fitted probabilities with the elicitated values. Facilitator feeds back two implied probabilities (10^th^ and 90^th^ percentiles) from the fitted distribution.]

Does the shape and distribution of this graph represent what you truly believe? (*yes/no*)

[If yes, proceed. If no, invite them to vary some values in order to fit others that are believed to be more pivotal. Re-fit a new distribution and feedback *until* vet believes the distribution is an accurate reflection of his belief.]

Imagine now that these *same* 100 chronically infected cows all received,

*intra-mammary dry cow therapy PLUS a systemic antibiotic regime of your choice also at the point of drying off*

instead of just intra-mammary dry cow therapy alone.

How likely is it that you would cure *less* cows by doing this? In other words, what is the probability that by giving systemic antibiotic as well as intra-mammary DCT you could make things worse? Recall that by answering “zero” that means you are *certain* you *won’t* cure less cows, by answering “one” that means you are *certain* you *will* cure less cows; you can answer anywhere between (and including) 0 and 1.

From now on we are going to assume that giving systemic antibiotic as well as intra-mammary DCT *cannot* make things worse, i.e. you can only cure the same number *or more* cows by giving systemic antibiotic as well as intra-mammary DCT.

Do you have any questions? (*yes/no*) If yes, proceed. If no, clarify

Consider the chronically infected cows that *are not* possible to cure with your intra-mammary DCT. Imagine there are100 of these cows.

Imagine what would have happened if these cows (that can’t be cured by intra-mammary DCT) instead of just receiving intra-mammary DCT had received instead:

*intra-mammary dry cow therapy PLUS a systemic antibiotic regime of your choice also at the point of drying –off*

Do you understand what we are now considering? (*yes/no*) If yes, proceed. If no, clarify

Some of these cows may be “cured” and calve in *not* infected, but you cannot be expected to know exactly how many may have cured.

[Now elicit the range, median and two further judgements. Fit probability distributions, produce graphs and feedback (as previously) using SHELF until vet is satisfied the pdf is a true reflection of their belief].

What effect do you think systemic antibiotics and intra-mammary DCT has in comparison to just intra-mammary DCT alone? Improves cure rate, no effect on the cure rate, worsens the cure rate? [Coherency check]

Do you feel this questionnaire has evaluated your beliefs about the efficacy of intra-mammary DCT in comparison to systemic antibiotics plus DCT? If not, are there any other comments you would like to make?

Please comment on whether or not you think the questions were clear?

Describe your clinical approach to prescribing systemic antibiotic for dairy cows at drying-off. (if ever used; drugs used, dose rate/regime/clinical situation). Does your practice have a policy about this?
